# Neural Correlates of Executed Compared to Imagined Writing and Drawing Movements: A Functional Magnetic Resonance Imaging Study

**DOI:** 10.3389/fnhum.2022.829576

**Published:** 2022-03-18

**Authors:** Alexander Baumann, Inken Tödt, Arne Knutzen, Carl Alexander Gless, Oliver Granert, Stephan Wolff, Christian Marquardt, Jos S. Becktepe, Sönke Peters, Karsten Witt, Kirsten E. Zeuner

**Affiliations:** ^1^Department of Neurology, University of Kiel, Kiel, Germany; ^2^Department of Radiology and Neuroradiology, University of Kiel, Kiel, Germany; ^3^Schreibmotorik Institut e.V., Heroldsberg, Germany; ^4^Department of Neurology, Evangelical Hospital Oldenburg and Research Center Neurosensory Sciences, Carl von Ossietzky University, Oldenburg, Germany

**Keywords:** writing network, kinematic writing parameters, motor imagery, sensorimotor cortex, premotor cortex, cerebellum

## Abstract

**Objective:**

In this study we used functional magnetic resonance imaging (fMRI) to investigate whether motor imagery (MI) of handwriting and circle drawing activates a similar handwriting network as writing and drawing itself.

**Methods:**

Eighteen healthy right-handed participants wrote the German word “*Wellen”* and drew continuously circles in a sitting (vertical position) and lying position (horizontal position) to capture kinematic handwriting parameters such as velocity, pressure and regularity of hand movements. Afterward, they performed the same tasks during fMRI in a MI and an executed condition.

**Results:**

The kinematic analysis revealed a general correlation of handwriting parameters during sitting and lying except of pen pressure during drawing. Writing compared to imagined writing was accompanied by an increased activity of the ipsilateral cerebellum and the contralateral sensorimotor cortex. Executed compared to imagined drawing revealed elevated activity of a fronto–parieto-temporal network. By contrasting writing and drawing directly, executed writing induced an enhanced activation of the left somatosensory and premotor area. The comparison of the MI of these tasks revealed a higher involvement of occipital activation during imagined writing.

**Conclusion:**

The kinematic results pointed to a high comparability of writing in a vertical and horizontal position. Overall, we observed highly overlapping cortical activity except of a higher involvement of motor control areas during motor execution. The sparse difference between writing and drawing can be explained by highly automatized writing in healthy individuals.

## Introduction

Handwriting is a highly skilled motor task involving a complex and highly trained motor network most likely associated with the activity of the contralateral primary sensorimotor area, the posterior parietal cortex with the superior and inferior parietal lobule, the lateral premotor cortex and ipsilateral to the cerebellum (Horovitz et al., 2013; Planton et al., 2013). In the majority of neuroimaging studies participants have been asked to write (Purcell et al., 2011; Segal and Petrides, 2012; Horovitz et al., 2013; Planton et al., 2013, 2017; Yuan and Brown, 2015). One exception is Planton’s study (2017), in which he compared writing to drawing shapes as a non-linguistic, non-stereotyped manual motor task with similar motor complexity. Both tasks recruited an overlapping network, but the writing task induced a specific left lateralization profile in the superior premotor cortex close to Exner’s area that Roux et al. (2009) named as the graphemic/motor frontal area (GMFA). The dorsal premotor area, the superior parietal cortex, the intraparietal sulcus and the right posterior cerebellum were engaged during writing and drawing. In a more recent study, functional connectivity between the Exner’s area and the right cerebellum was greater in females compared to males during a Chinese handwriting task and drawing nonsense symbols (Yang et al., 2020).

Handwriting is an automatized task, but the motor program needs to be updated continuously to produce the correct strokes. This is in contrast to circle drawing, which is—as opposed to Planton’s task of drawing shapes—an automatized and stereotyped drawing movement pattern. A possibility to further characterize and distinguish these different movement patterns consists in the kinematic writing analysis (Marquardt and Mai, 1994; Marquardt et al., 1999). Combining the kinematic writing analysis and functional imaging provides the opportunity to deepen our understanding of the neuronal pathways. In this work we aimed to investigate the different neuronal activation patterns underlying both tasks, as a proof of concept to later investigate the disruptions within this network as for example in patients with writer’s cramp, the most common task specific dystonia with muscle co-contraction during writing.

One attractive concept to avoid non-specific muscle activations during fMRI and therefore to assess the writing network is motor imagery (MI). MI is an internal movement simulation and implies the visualization of movement performance without executing it (Jeannerod, 1995; Jeannerod and Decety, 1995; Hétu et al., 2013; Kilintari et al., 2016) and is thus considered as the conscious representation of a non-movement (Kilintari et al., 2016). MI activates similar areas as executed movements such as prefrontal areas (Hétu et al., 2013), the premotor cortex as a center for movement planning and preparation of movements (Hoshi and Tanji, 2007) and association cortices (Jeannerod, 1995; Jeannerod and Decety, 1995; Decety, 1996; Hétu et al., 2013). In a meta-analysis including fMRI and PET studies, pure MI (imagine a given movement focusing on motor aspects) has been differentiated from kinesthetic (feel the movement) or visual imagery (to self-visualize the execution of the movement) (Hétu et al., 2013). Pure MI of upper limb movements recruited a fronto-parieto-cerebellar network (Hétu et al., 2013) similar to the writing network mentioned above. However, studies using specifically MI of writing are sparse and included writing ideograms (Seitz et al., 1997), morphograms and syllograms (Tokunaga et al., 1999) or writing the name of an object (Harrington et al., 2007). MI offers the possibility to avoid the confounding factor of dystonic co-contractions during writing in patients with writer’s cramp (Castrop et al., 2012). To our knowledge, there are only two studies dealing with writer’s cramp patients performing MI during fMRI. The first compared kinesthetic MI of drawing simple geometric figures and observing hands drawing identical geometric figures (Castrop et al., 2012); The second study investigated writing and sharpening a pencil (Delnooz et al., 2012). Writing and stereotyped drawing have not been compared in this population. On the other hand, the exact characterizations of executed and imagined motor performance are required to understand the differences or similarities between those tasks and the abnormal pathophysiology of the neuronal network in writer’s cramp patients.

In this preparatory study we investigated MI of writing and more stereotyped drawing tasks in healthy participants with the purpose to transfer this paradigm to writer’s cramp patients in future experiments.

We designed a paradigm with four conditions (writing and stereotyped drawing as well as both tasks during MI) performed by healthy individuals during fMRI recordings. By using the kinematic writing analysis on both tasks overtly performed outside the scanner, we compared the movements in an everyday writing and in the supine fMRI position. Since the majority of writing paradigms during functional imaging fails to simulate daily routine writing resulting in a lack of ecological validity (Karimpoor et al., 2018). With this study we aimed to answer the following questions:

i. Does MI of writing activate the same network as executed writing?
ii. Does MI of circle drawing activate the same network as executed circle drawing?
iii. Does writing, as a more complex task, cause an increased BOLD response in the premotor cortex, primary motor cortex and the cerebellum compared to a stereotyped movement as circle drawing.
iv. Does circle drawing cause more activation in the prefrontal and parietal cortices compared to writing?

## Methods

### Participants

We investigated one group of eighteen healthy participants (9 women) with a mean age of 49.94 ± SD 13.72 years (range: 23–64). They had no history of neurological or psychiatric illnesses and gave written informed consent to take part in the study. All subjects were right-handed (laterality quotient: 91.52 ± SD 10.12, range: 70–100) according to the Oldfield handedness test (Oldfield, 1971). We had to exclude two participants because of movement artifacts. The study was approved by the ethics committee of the University Medical Faculty in Kiel and was conducted in full accordance with the Declaration of Helsinki.

### Experimental Design

#### Kinematic Writing Analysis

Prior to scanning, all subjects wrote the German word *Wellen* (waves) in cursive mode several times over 60 s on a graphic tablet. “Wellen” was chosen as a simple word without kinematic breaks and minimal hand movements. In addition, they drew circles for 60 s in a stereotyped manner. Participants received no visual feedback during writing or circle drawing to allow fast, automated hand movements. Additionally, the tasks were performed mainly with finger and small hand movements at the most to avoid movement artifacts. They performed the task once while sitting and once in a horizontal position without looking at their hand. The purpose was that all subjects got familiar with the task before performing them in the scanner. In addition, the recording of the kinematic parameters allowed us to determine whether the writing and drawing in a supine position are comparable to those performed in a vertical sitting position.

Drawing and writing movements were recorded using a pressure-sensitive digitizing tablet and an appendant ballpoint pen (WACOM Intuos 3, A4; Wacom Europe, Düsseldorf, Germany). The position of the pen tip was recorded with a sample frequency of 200 Hz and a spatial resolution of 0.05 mm (actual accuracy 0.1 mm), stored on a computer and analyzed using the CSWin 2016 software (MedCom, Munich, Germany). For the kinematic analyses, the parameters pen pressure, frequency (frequency of up and down strokes) and automation index NIV (number of inversions in velocity per stroke) (Marquardt et al., 1999) were used. The NIV is a measure of movement fluency and automation (Marquardt et al., 1999). Automation of handwriting movements typically presents with smooth and single peaked velocity profiles. The NIV describes the number of directional changes in velocity during writing. More precisely, it corresponds to the number of peaks in the velocity profile associated with a single up or down stroke and is estimated by counting the number of zero-crossings in the corresponding acceleration profile. In healthy subjects, the value is 1, reflecting a velocity profile that exhibits just one peak. If the movements are not fluently, but disturbed the NIV is > 1.

#### Functional Magnetic Resonance Imaging Paradigm

**FIGURE 1:**
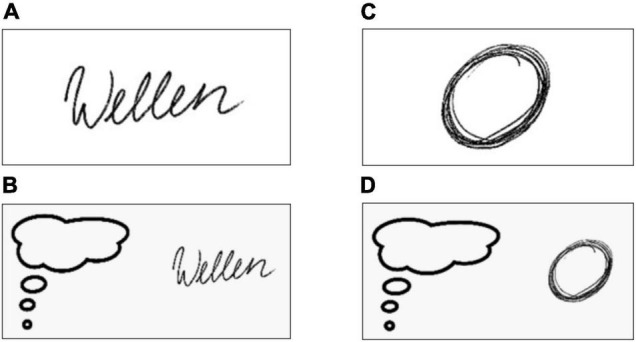
Instruction images for the four conditions of the fMRI task. The subjects wrote the German word “Wellen” **(A)** or imagined to write this word **(B)**. In parallel, the subjects drew circles **(C)** or imagined to draw circles **(D)**.

To ensure a stable performance during scanning, prior to scanning all the participants were instructed how to correctly perform the tasks. They were also familiarized with the visual stimuli driving the tasks within the scanner (Figure 1). During fMRI the participants laid in a comfortable position with bent knees held up by a cushion. The head was fixed tightly in the MRI coil, the arm was supported with foamed material to avoid arm movements. A MRI compatible tablet with 8.4″ from DMC Co., Ltd. (Tokyo, Japan) was positioned on their tights. The writing was performed with a plastic ballpoint. The paradigm included 4 conditions each presented 10 times in a pseudorandomized order. We divided the 40 blocks into 2 sessions to avoid fatiguing. Between the 2 sessions there was a break of 10 min, while we acquired anatomical images [T1, T2-weighted fluid attenuated inversion recovery (FLAIR)]. Supplementary Figure 1 depicts the exact temporal pipeline of the fMRI paradigm.

During each condition (executed writing, imagined writing, executed drawing, imagined drawing) a visual stimulus was presented to the subjects for the entire duration of that condition to ensure, that they were always aware of the instruction (Figure 1). In the *writing* condition the subjects wrote the German word *Wellen* (waves) in cursive mode for 20 s on a MRI compatible tablet in their usual writing velocity (Figure 1A). In accordance with the concept of kinesthetic MI, in the MI condition participants were asked to imagine writing the German word *Wellen* for 20 s (Figure 1B). More specifically, they were instructed to actually imagine the process of writing as if they executed the task and to concentrate on the movement of the lower arm and hand. The third and fourth condition included drawing circles (Figure 1C) and imagining drawing circles (Figure 1D) were performed in a similar way, again for 20 s each. Each task block was followed by a 10 s break visually marked by the presentation of a central fixation cross. During the MI conditions and breaks participants were instructed to continue holding the pen, but to move the forearm away from the tablet. As we plan to apply this paradigm in patients with writer’s cramp, this posture is intended to minimize involuntary activity.

#### Control of Muscle Activity During the Motor Imagery Tasks

To exclude involuntary writing or drawing during the MI tasks we recorded the muscle activity using an MRI compatible electromyogram (EMG) (BrainAmp ExG MR, Brain Products GmbH, Gilching, Germany) with two adjacent surface electrodes on the flexors and two on the extensors of the right forearm, resulting in two EMG-derivations, one for each of the corresponding muscular compartments of the forearm. The EMG signal was recorded using BrainVision Recorder and observed online via BrainVision RecView (Brain Products GmbH, Gilching, Germany). In addition, an online observation of the muscle activity was realized via the BrainVision RecView (Brain Products GmbH, Gilching, Germany).

### Magnetic Resonance Imaging Data Acquisition and Preprocessing

Anatomical and functional images were acquired in the Neurocenter at Kiel University Hospital with a 3T whole-body MRI scanner (Ingenia CX 3T, Philips, The Netherlands) provided with a 32-channel head coil. For stimulus presentation, a visual system of NordicNeuroLab (Bergen, Norway) with a resolution of 800 × 600 px was used. For functional MRI a whole-brain echo planar imaging (EPI) sequence with the following parameters was used: Repetition time (TR) = 2 ms, echo time (TE) = 30 ms, field of view (FOV) = 192 × 192 mm^2^, flip angle (FA) = 90°, matrix = 64 × 64, slices = 48, slice thickness = 3.0 mm, and inter-slice gap = 0.3 mm and 300 volumes over the experimental scan time of 10 min twice, interspersed by the recording of the structural images (Supplementary Figure 1). This led to 600 volumes and a total experimental scan time of 20 min. The axial slices were acquired parallel to the anterior-posterior plane. For all subjects, additional three-dimensional (3D) T1-weighted gradient echo MRI scans with sagittal volume excitation were acquired with the following parameters: TR = 6.7 ms, TE = 3.1 ms, FOV = 270 × 253 mm^2^, flip angle = 9°, slices = 170, matrix = 244 × 230, voxel size = 1.1 × 1.1 × 1.2 mm^3^. An additional FLAIR sequence was performed to screen for structural abnormalities.

For image preprocessing and functional analysis, the SPM12 (Release 7219) software package Wellcome Department of Imaging Neuroscience, London)^1^ as well as Matlab Version 9.7 (R2019b) (MathWorks Inc., Natick, MA, United States) were used. In a first step of the preprocessing, all functional EPI images were realigned to correct for subjects’ movements during the scanning. Next the anatomical T1-weighted images were spatially normalized to the standard coordinates of the Montreal Neurological Institute (MNI) space. The SPM normalization procedure (implemented as SPM-segmentation) utilizes a non-linear transformation and includes a bias intensity correction of the structural T1 images. Further the realigned EPI images (3 × 3 × 3.3 mm^3^) were co-registered with the corresponding bias corrected individual T1-weighted image (rigid-body transform) and then normalized to MNI space using the same non-linear transformation as estimated in the structural image normalization procedure. This alignment and normalization steps provide the functional images in a spatial format suitable for a voxel-wise analysis of the BOLD time-series and subject independent statistical comparisons. Finally, we smoothed the functional MRI data with a Gaussian kernel filter of 8 mm full-width at half-maximum (FWHM).

### Functional Analysis (Functional Magnetic Resonance Imaging)

The first level analysis of the paradigm was performed using a General linear model (GLM) with four regressors of interest: writing, imagined writing, drawing, imagined drawing modeling the experimental timing of the four different tasks. As regressors of no interest the six realignment parameters were included. To prevent movement related false positive activations, we checked the design orthogonality of our first—level models with these realignment parameters in SPM. Here we could not find any important correlations between the block-task events and the six movement regressors. We then computed contrast images for the conditions writing, imagined writing, drawing, imagined drawing, as well as differences between those (writing—imagined writing, drawing—imagined drawing, writing—drawing and imagined writing—imagined drawing).

On the second level, we used separate SPM models to compute the main effect of conditions by means of one sample *t*-tests with the respective first level contrast images.

In a first step, we analyzed BOLD signal changes on whole brain level concerning all clusters with an FWE corrected *p*-value below 0.05 as significant. As results the cluster sizes, the corresponding cluster *p*-value, the coordinates of the cluster peaks as well as further relevant local maxima within these clusters are reported.

In a second step, we limited our analysis to a handwriting network published by Planton et al. (2013) including the left hemisphere parts of the superior frontal gyrus, the primary motor and somatosensory cortex, the supplementary and pre-supplementary area, parts of the ventral premotor area and inferior frontal gyrus, the superior parietal lobule and parts of the posterior inferior temporal cortex. On a subcortical level, the left thalamus and the left putamen were included. On the right hemisphere, we included the anterior and posterior cerebellar lobes, parts of the superior frontal gyrus and the inferior parietal lobule. A detailed definition of the regions of interests (ROIs) are presented in the Supplementary Table 1. After aggregation all 12 predefined ROIs, 26,650 voxels (∼791.5 cm^3^) were included in the region of interest analysis. Again, clusters with an FWE corrected *p*-value below 0.05 were considered significant. As results the cluster sizes, the corresponding cluster *p*-value, the coordinates of the cluster peaks are reported.

In case of the contrasted effects (writing > imagined writing, drawing > imagined drawing, writing > drawing and imagined writing > imagined drawing and vice-versa) on second level, we used the AAL 3 toolbox to determine which proportions of each significant cluster belongs to which AAL region, as only concerning the label of the cluster peak can lead to misleading interpretations of the results (Rolls et al., 2020).

Finally we conducted conjunction contrasts (method conjunction null) between executed writing and imagined writing, executed circle drawing and imagined circle drawing, executed writing and circle drawing and imagined writing and circle drawing in order to locate regions with common activations. To specify the results of the conjunction contrasts, we performed again a ROI analysis with the above mentioned masks. The results are reported with a FWE corrected *p*-value below 0.05.

## Results

### Performance of the Kinematic Writing Analysis

As mentioned in the methods section, all subjects performed the writing and the repetitive circle drawing task outside the scanner while sitting and in a lying position for 60 s each. The mean values of pressure, frequency and NIV are reported in Table 1. Except pressure for circle drawing in a sitting and lying position, all kinematic parameters are highly correlated and therefore comparable independently of the actual position of the subject. The record of a complete kinematic dataset of all subjects during the fMRI sessions failed due to technical problems, thus we excluded this part of our data from our analysis to avoid statistical effects driven by artifacts.

**TABLE 1:** Parameter of the kinematic analysis for writing the German word “Wellen” or drawing circles in a sitting and in a horizontal position and the correlation between the conditions.

	Writing “*Wellen*”				Drawing circles			
	Mean ± SD sitting	Mean ± SD lying	r	*t*-value	Mean ± SD sitting	Mean ± SD lying	r	*t*-value
Pressure (N)	1.75 ± 0.49	1.60 ± 0.39	0.84*	6.06	1.13 ± 0.37	1.18 ± 0.30	0.46	2.02
Frequency (Hz)	4.08 ± 0.71	3.85 ± 0.68	0.87*	6.82	3.43 ± 0.82	3.16 ± 0.81	0.83*	5.85
NIV	1.15 ± 0.15	1.14 ± 0.14	0.85*	6.29	1.17 ± 0.36	1.24 ± 0.77	0.95*	12.07

*p < 0.0001.

### Functional Imaging Results

#### The Handwriting Network

**FIGURE 2:**
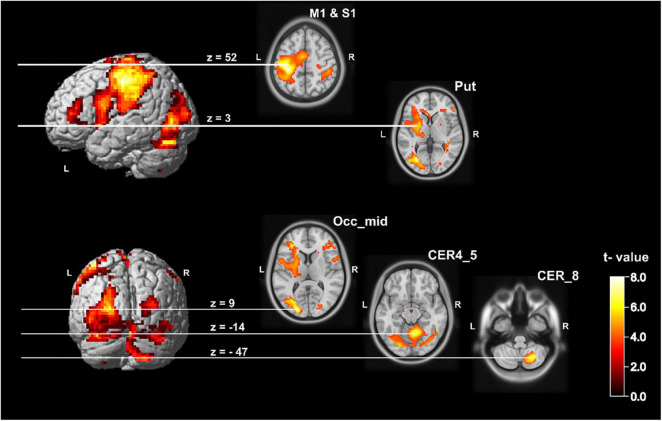
Statistical map showing group brain activation related to the handwriting condition. It shows the five relevant clusters. On whole brain level: left postcentral gyrus with a cluster peak at [−33 −31 52], middle occipital gyrus left with a cluster peak at [−21 −88 9], right posterior cerebellum (Lobule VIII) with cluster peak at [27 −64 −47] and the right anterior cerebellum (Lobule IV and V) with a cluster peak at [9 −55 −14] (*p* < 0.05, FWE corrected). The left putamen with a cluster peak at [−27 −4 3] and the left precentral gyrus with a cluster peak at [−51 2 26] became significant on *p* < 0.05 (FWE corrected) within the ROI analysis (see Supplementary Table 1). CER, cerebellum; M1, primary motor cortex; Occ_mid, occipital middle gyrus; Put, putamen; S1, primary somatosensory cortex.

The analysis of the handwriting of the sixteen subjects on whole brain level resulted in a significant increased left sided BOLD signal postcentral with a cluster peak at [−33 −31 52], a right hemisphere posterior and anterior cerebellar cluster (cluster peak within the cerebellum VIII [27 −64 −47]; cluster peak in lobes IV and V [9 −55 −14]. Additionally, we found a cluster within the left middle occipital gyrus with a cluster peak at [−21 −88 9]. The ROI analysis (for details see Methods section and Supplementary Table 1) additionally revealed an increased BOLD signal in handwriting within the left putamen [−27 −4 3] and the precentral gyrus [−51 2 26]. All results are significant on cluster level after FWE correction on *p* < 0.05. A detailed overview of activated clusters and relevant local maxima is given in Supplementary Table 2 and the network is depicted in Figure 2.

#### Differences and Similarities Between Handwriting and Circle Drawing

**FIGURE 3:**
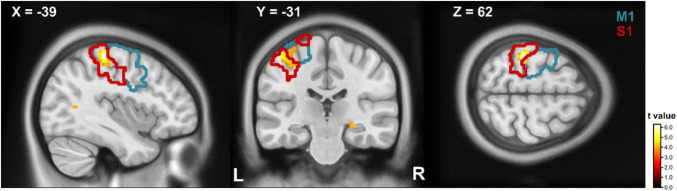
T-maps for writing compared to drawing. Parts of the precentral (blue contour) and postcentral (red contour) gyrus showed a stronger BOLD response on writing compared to drawing. The contours refer to the respective regions of the AAL atlas. Clusters are significant within the ROI analysis after FWE-correction on *p* < 0.05. M1, primary motor cortex; S1, primary somatosensory cortex.

In the ROI analysis we found a stronger neural activation for writing compared to drawing (writing > drawing) in a cluster of the left postcentral gyrus with a cluster peak at [−39 −31 62] (Figure 3). According to the AAL atlas, 72.2% of the clusters’ voxel are located inside the postcentral and 27.8% in the precentral gyrus. The conjunction analysis involved several regions of the motor network including the left precentral gyrus, left supplementary motor area, the leftsided putamen and thalamus, the vermis, the cerebellum bilateral, and the inferior parietal area (Supplementary Table 3). The contrast drawing compared to writing (drawing > writing) revealed no difference in BOLD signal amplitude.

##### Differences and Similarities Between Motor Imagery of Writing and Drawing

MI of handwriting exhibited significant clusters on whole brain level in the left inferior parietal cortex, the left precentral cortex and the left superior occipital cortex Table 2 . The ROI analysis additionally revealed a significant cluster in the left superior parietal lobe. MI of circle drawing induced no significant BOLD activity. The conjunction analysis located regions with common activations in the left putamen and the right cerebellum.

**TABLE 2:** Results of the second level contrast imagined writing.

AAL label	Side	MNI coordinates of cluster peak			*t*-value of cluster peak	*p*-value of cluster peak	Cluster size	*p*-value on cluster level
		x	y	z				
**Imagined writing**								
**A: whole brain analysis**								
Parietal_Inf	L	−33	−46	45	7.37	0.018	345	0.002
Precentral	L	−51	2	36	5.57		204	0.017
Occipital_Sup	L	−24	−82	32	5.47		159	0.036
**B: ROI-analysis^+^**								
Parietal_Sup	L	−33	−64	56	5.52	0.048	87	0.043

*The AAL labels of the cluster peaks on whole brain level (A) are reported together with the cluster size and the t-value of the respective cluster peak and the cluster p-value (p < 0.05, FWE corrected). In section (B) the clusters peaks are listed, that became significant on p < 0.05 (FWE corrected) on cluster level in the ROI analysis (see Supplementary Table 1).*

*^+^For the ROI analysis, only clusters are reported, that became not significant on FWE corrected whole brain level (p < 0.05).*

##### Differences and Similarities Between Executed Handwriting and Motor Imagery of Handwriting

**FIGURE 4:**
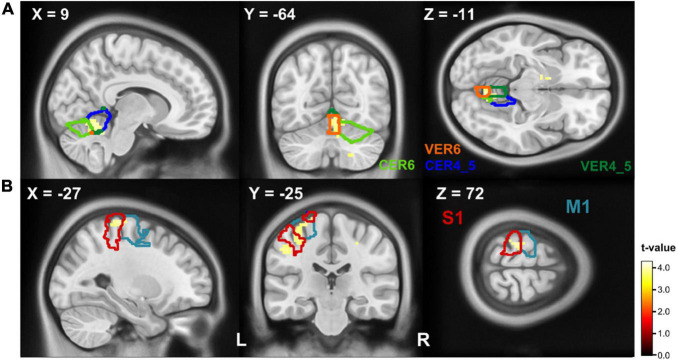
T-maps of BOLD differences in the contrast writing > imagined writing. The contours (referring to the AAL atlas) depict the cluster’s participating regions: **(A)** writing activated parts of the ipsilateral cerebellum (Lobule IV and V, blue contour; Lobule VI, light green contour; Vermis IV and V, dark green contour; Vermis VI, orange contour) more than the imaged writing. **(B)** Parts of the precentral (blue contour) and postcentral (red contour) gyrus showed a stronger BOLD response on writing compared to imagined writing. Clusters are significant within the ROI analysis after FWE-correction on *p* < 0.05. CER, cerebellum; M1, primary motor cortex; S1, primary somatosensory cortex; VER, vermis.

The comparison of executed handwriting and imagined handwriting showed an increased BOLD signal for handwriting compared to imagined writing in a cluster that covers parts of the ipsilateral anterior cerebellum (Lobule IV and V, Lobule VI) and in the cerebellar vermis (Vermis IV and V, Vermis VI) (Figure 4A). The ROI analysis resulted in a second significant cluster of increased BOLD signal covering parts of the pre- and postcentral gyrus (Figure 4B). A detailed distribution of the cluster parts in the respective AAL region can be found in Table 3. The conjunction between executed writing and imagined writing involved the left putamen and the right cerebellum.

**TABLE 3:** Results of the second level contrast writing > imagined writing.

AAL label	Side	MNI coordinates of cluster peak			*t*-value of cluster peak	Cluster size	*p*-value on cluster level
		x	y	z			
**Writing > imagined writing**							
**A: whole brain analysis**							
	R	9	−64	−11	4.30	187	0.010
Cerebellum IV and V (44.9%)							
Vermis IV and V (18.2%)							
Vermis VI (13.9%)							
Cerebellum VI (10.2%)							
**B: ROI analysis^+^**							
	L	−27	−28	72	4.05	80	0.033
Postcentral (50%)							
Precentral (50%)							

*The percentages behind the AAL labels describe the parts of the cluster on the respective AAL label corresponding to the AAL 3 toolbox. The AAL labels of the cluster peaks on whole brain level (A) are reported together with the cluster size and the t value of the respective cluster peak and the cluster p-value (p < 0.05, FWE corrected). In section (B) the clusters peaks are listed, that became significant on p < 0.05 (FWE corrected) on cluster level in the ROI analysis (see Supplementary Table 1).*

*^+^For the ROI analysis, only clusters are reported, that became not significant on FWE corrected whole brain level (p < 0.05).*

**FIGURE 5:**
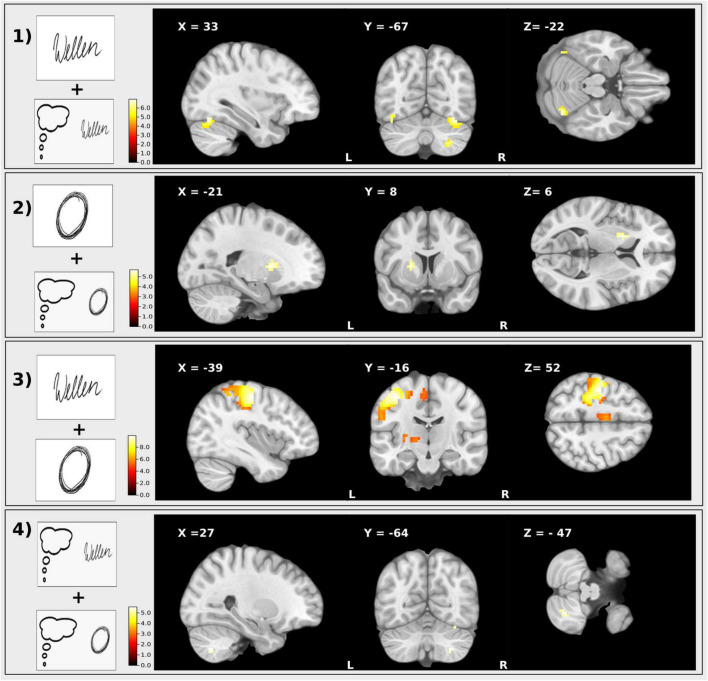
T-maps of the results of the different conjunction contrasts. (1) Conjunction: Executed writing and imagined writing, (2) Executed drawing and imagined drawing, (3) Executed writing and drawing, (4) Imagined writing and drawing. Clusters are significant within the ROI analysis after FWE-correction on *p* < 0.05.

Imagined writing compared to writing (imagined writing > writing) displayed no differences in BOLD activity Figure 5.

#### Differences and Similarities Between Executed Drawing and Imagined Drawing

Executed drawing compared to the imagined drawing led to an increase of activation in the bilateral frontal lobe (Table 4). The main cluster peak laid in the medial part of the right superior frontal gyrus, additional cluster peaks included the medial part of the left superior frontal gyrus and the bilateral anterior cingulate gyrus. We detected an additional BOLD signal increase in the left superior temporal gyrus, the bilateral medial cingulum and in a cluster covering parts of the right angular gyrus as well as the inferior and superior parietal lobule. We found no significant increased BOLD signal for imagined drawing compared to drawing (imagined drawing > drawing). The conjunction analysis involved the left sided putamen and the right cerebellum.

**TABLE 4:** Results of the second level contrast drawing > imagined drawing on whole brain level.

AAL label	Side	MNI coordinates of cluster peak			*t*-value of cluster peak	Cluster size	*p*-value on cluster level
		x	y	z			
**Drawing > imagined drawing**							
**Whole brain analysis**							
	L/R	9	50	12	5.58	345	0.000
Frontal_Sup_Med_R (30.8%)							
Frontal_Sup_Med_L (22.9%)							
ACC_pre_R (17.8%)							
ACC_pre_L (15.5%)							
	L	−57	−34	12	5.20	134	0.016
Temporal_Sup (63.4%)							
Heschl (10.5%)							
	L/R	−15	−28	39	5.06	264	0.001
Cingulum_Mid_L (32.2%)							
Cingulum_Mid_R (27.7%)							
Precuneus_R (17.4%)							
	R	39	−70	45	4.88	148	0.01
Angular_R (54.1%)							
Parietal_Inf_R (27.0%)							
Parietal_Sup_R (18.9%)							

*The percentages behind the AAL labels describe the parts of the cluster on the respective AAL label corresponding to the AAL 3 toolbox. The AAL labels of the cluster peaks on whole brain level are reported together with the cluster size and the t value of the respective cluster peak and the cluster p-value (p < 0.05, FWE corrected).*

#### Differences and Similarities Between the Motor Imagery of Writing and Motor Imagery of Circle Drawing

On whole brain level, neural activation was stronger for imagined writing compared to imagined drawing (imagined writing > imagined drawing) in a cluster of the right occipital gyrus ([27 −82 29]; *k* = 122, *p* < 0.05, FWE corrected on cluster level). According to the AAL atlas, 71.1% of the clusters’ voxel laid inside the middle and 27.8% in the superior occipital gyrus. There were no differences between imagined drawing compared to imagined writing (imagined drawing > imagined writing). The conjunction analysis involved the left putamen and the right cerebellum.

## Discussion

In this proof-of-concept study we compared the neural correlates of executed and imagined writing and drawing in healthy participants. Since there is an assumed lack of ecological validity of writing in the scanner (Karimpoor et al., 2018), we evaluated the kinematic writing parameters of both tasks prior to scanning in a usual writing situation (sitting) and a scanning (supine) position. The velocity of writing and circle drawing and the number of inversions as a measurement of movement regularity were highly correlated between sitting and lying, while the pressure showed such a relation only for writing and not for circle drawing. Thus, in our paradigm, writing and circle drawing were comparable to writing in an everyday (sitting) situation. One important difference is, however, that during scanning, due to the visual instructions, the participants had no visual feedback and may compensate the absence of a visual control by enhanced sensorimotor control and feedback mechanisms.

The neuroimaging data of the present fMRI study showed that (1) executed handwriting produced similar neural cortical activation patterns as drawing, but a stronger BOLD signal in the postcentral and precentral gyrus; (2) imagined writing exhibited a significant cluster precentral, in the left superior parietal and superior occipital cortex (Table 2); (3) writing compared to imagined writing was associated with increased BOLD signal in the ipsilateral cerebellum and the contralateral primary sensorimotor area; however, the right cerebellum, the left putamen and the fusiform gyrus were involved in the conjunction analysis; (4) executed compared with imagined stereotyped circle drawing resulted in an increased activation of a fronto-parieto-temporal network, but the right cerebellum and the left putamen passed the conjunction analysis; (5) imagined writing in contrast to drawing induced an elevated signal in the occipital lobe, the right cerebellum and left putamen were interconnected.

### The Writing Network

The regions we were able to identify during writing (Figure 2) are known for their role in the writing network (Horovitz et al., 2013; Planton et al., 2013). The somatosensory cortex has been related to the sensory control such as pen grasping and limb movements during writing. The ipsilateral anterior and posterior cerebellar activity is typically assigned to the processes of motor control including the motor output during writing (Katanoda et al., 2001; Purcell et al., 2011; Segal and Petrides, 2012; Horovitz et al., 2013; Planton et al., 2013, 2017; Karimpoor et al., 2018). However, the exact function of the anterior and posterior cerebellum in handwriting is not entirely clear (Planton et al., 2013), and is regarded as not specific for writing (Planton et al., 2017).

In addition to the cerebellar activation, we found another non- specific writing area located in the left inferior parietal lobe that has been attributed to manual tasks such as handwriting (Planton et al., 2017).

Finally, we observed activation of the left middle occipital lobe and the left putamen during writing. Since the instructions during the task were visually presented, an activation of a visual area is consistent with processing of the stimuli. However, we have to consider that visual feedback as a substantial element of motor control requires occipital activation during writing. Putaminal activation has been previously reported during writing (Purcell et al., 2011; Horovitz et al., 2013; Planton et al., 2013; Bisio et al., 2016), but as it is not a writing specific region, the activation has been explained by the motor nature of the task. Recently it has been suggested that the putamen is involved in integrating the information of letter shapes with planning and execution of handwriting (Bartoň et al., 2020).

### Differences and Similarities Between Writing and Drawing

Consistent with the motor complexity of executed writing compared to stereotyped circle drawing, writing revealed increased BOLD signal in the left primary sensorimotor area. The left lateralization during handwriting is in line with the literature (Menon and Desmond, 2001; Planton et al., 2017; Yang et al., 2020). In contrast to handwriting, circle drawing has been considered a more stereotyped task (Marquardt et al., 1999). Motor network activation during stereotyped circle drawing was similar as during writing. One possible explanation is that in our healthy cohort, the degree of automaticity during writing the word *“Wellen”* was similar to that of circle drawing, during the lying position in the MRI scanner. In our study, MI of handwriting was interconnected with increased occipital activity. This result may be attributed to increased visuospatial processing during writing (Fortin et al., 2002; Waberski et al., 2008).

### Cortical Patterns During Movement Execution and Motor Imagery

#### Executed vs. Imagined Handwriting

##### The Sensorimotor Cortex During Writing

There are different modalities how to perform MI. According to the meta-analysis form Hétu et al. (2013) pure MI implies that a person imagines himself to pick up a pen for writing and concentrates on the motoric aspects of that movement. While kinaesthetic MI involves to feel the actual movement, the muscles and the process of writing, visualized MI consists of self-visualizing the execution of handwriting. In our study we asked the participants to concentrate on the movement of the forearm as if he/she would actually perform the actual task. In that regard, our instructions corresponded to the kinaesthetic instructions (Hétu et al., 2013).

The regions that were activated by MI in our study were similar to the activity during executed handwriting, although the latter task revealed a clearly increased BOLD signal in the left primary sensorimotor area. This finding is consistent with the motor complexity of executed writing compared to imagined handwriting. The activity in the parietal gyrus most likely results from the sensory input of the pen during writing, which is activated during both, imagined and executed writing, especially since our participants were asked to keep the pen in their hands during MI. As we monitored each muscle activation online during the scanning period, we excluded any involuntary muscle activation during MI as a confounding factor. Taken together, our results showed similar activity during both tasks, except the increased activity in the sensorimotor cortex that is clearly related to the motor output during motor execution of writing.

##### The Role of the Cerebellum

One of our main questions was, whether writing and imagined writing are accompanied by the same cortical patterns (i). As mentioned above, the ipsilateral cerebellum is of great importance during writing and has been identified as part of the MI network as well. This finding was confirmed in the conjunction analysis, the cerebellum passed for the writing condition, circle drawing, imagined writing, and imagined circle drawing. While lesions in the cerebellum are known to impair MI (González et al., 2005; Battaglia et al., 2006; Grealy and Lee, 2011), cerebellar activity of the lobules VI, VII and the vermis have been reported during imagined writing (Hétu et al., 2013). Interestingly the contrast writing > imagined writing revealed increased activation of the ipsilateral cerebellum, including the segments IV, V, and VI.

Lobule V and lobule VI are known to be involved in the writing network (Planton et al., 2013) and the additionally observed area IV has been recently observed during a finger tapping task (Stoodley et al., 2012). Another area that showed increased BOLD activity in our study was the spinocerebellar vermis (Vermis IV and V, Vermis VI). The vermis has been shown to be active during eye and hand coordination (Ellermann et al., 1998; Inoue et al., 1998; Miall et al., 2000; Nitschke et al., 2005) which is a basic feature of handwriting. In summary, the motor complexity of executed writing combined with the necessary eye-hand coordination might best explain the increased cerebellar activation during movement execution.

#### Differences and Similarities of Executed and Imagined Circle Drawing

The drawing task was chosen to induce automatized stereotyped movements. Analyzing the neuronal activity revealed similar patterns during execution vs. imagination (ii), except for a few areas mainly known for their crucial role in the writing network.

##### Prefrontal Cortex: Superior Frontal Gyrus and Anterior Cingulate Cortex

Both regions, the bilateral superior frontal gyrus (Sugihara et al., 2006; Purcell et al., 2011; Planton et al., 2013) and the anterior cingulate cortex (Ceballos-Baumann et al., 1997; Purcell et al., 2011; Segal and Petrides, 2012; Horovitz et al., 2013; Yang et al., 2020) are associated with handwriting. While a left sided lateralization is considered to be specific for handwriting (Planton et al., 2017), a bilateral activation of the superior frontal gyrus has been attributed to precise manual tasks. The right superior frontal gyrus presents with increased activation during drawing a picture compared to MI of that task (Harrington et al., 2007).

The activity of the cingulum is not specific for circle drawing, but has rather been attributed to hand movements (Lotze et al., 1999; Yuan and Brown, 2015). It is also engaged during visuospatial imagery (Suchan et al., 2002). The anterior cingulate cortex is enabled during both, executed and the mental simulation of movements (Grèzes and Decety, 2001) and additionally during kinesthetic imagery (Kilintari et al., 2016), but these modalities have not been contrasted. Similar to the handwriting paradigm, the motor output is higher during the execution of stereotyped circle drawing. Therefore, in our study these regions were more active during executing rather than MI of circle drawing.

##### The Superior Temporal Gyrus

The superior temporal gyrus belongs to the writing network (Planton et al., 2013). Previous reports suggest that the bilateral superior/middle temporal gyri are in particular active while drawing (Rapp and Dufor, 2011). The elevated signal of the superior temporal gyrus in our sample is probably caused by general, unspecific movement execution (Kilintari et al., 2014).

##### The Fusiforme Gyrus

The fusiform gyrus was a region of common activation in the conjunction analysis for executed and imagined writing. The left inferior temporal region as part of the fusiforme gyrus is known to be part of the writing network. This area has also been named Visual Word Form Area and has been attributed to letter recognition (Cohen et al., 2000). Here, in our study letter recognition is mandatory during executed and imagined writing. However, there has been some discussion whether this area is part of the visual system and its activation may be vision-related (Planton et al., 2017).

##### The Parietal Lobe

The role of parietal lobe during movement execution (Wise et al., 1997; Fogassi and Luppino, 2005) and MI (Sirigu et al., 1996; Hétu et al., 2013) is well-described. There are three regions of the parietal lobe that have been active during executed compared to imagined stereotyped circle drawing and will thus be discussed further:

First, the posterior parietal cortex plays a major role in sensorimotor processing (Brownsett and Wise, 2010), the online control during manual activities (Desmurget et al., 1999; Tunik et al., 2005) and during visual guided activities (Mutha et al., 2011). In addition, the posterior parietal cortex is involved in complex, precise manual tasks. Consistent with the results of our study that showed increased activity in the parietal cortex during executed compared to imagined stereotyped circle drawing, Planton et al. (2017) suggested a bilateral parietal drawing area in the posterior part of the inferior parietal sulcus (Planton et al., 2017).

Second, the posteromedial part of the parietal lobe, the precuneus, plays a central role for a wide spectrum of highly integrated tasks. It is involved in visuo-spatial information processing (Cavanna and Trimble, 2006), it seems to be important for spatially guided behavior (Selemon and Goldman-Rakic, 1988) and in visual reaching of objects (Johnson et al., 1996). Thus, an involvement of this area is fundamental for accurate hand movements, which explains an increased activity during executed vs. imagined stereotyped circle drawing in our study.

Finally, located in the posterior part of the inferior parietal lobule the right angular gyrus is activated in a variety of tasks including attention and spatial cognition during motor execution (Seghier, 2013). Consistent with our results, the angular gyrus is involved during drawing (Yuan and Brown, 2015) and writing (Gubbay and de Klerk, 1995; Ardila, 2014; Planton et al., 2017).

In summary, the increased parietal activation in our sample can be explained by the complexity of executed movements and the online sensorimotor processing of movements. In our paradigm the visuomotor coordination is less relevant since the subjects had no visual feedback.

### Limitations

As a pilot study, we have to consider several limitations. First although we tried to simulate an everyday writing situation as best as possible, the posture in a MRI remains always unnatural. In addition, there was no visual feedback during all tasks in our paradigm, since our participants looked at the optical stimuli. Furthermore, although writing kinematics have been analyzed prior to scanning, there is no analysis of movements during scanning. Nevertheless, since these drawbacks of our paradigm affect all conditions, their knowledge is less important for the comparison of the different conditions. Besides that, finding an optimal control task for writing is difficult. As we focused on motor aspects, in particular the cognitive and linguistic aspects are neglected in this work.

## Conclusion

Our study identified the interacting neuronal regions during a writing and drawing task and contrasted these activations with corresponding MI tasks. We observed highly overlapping neural networks between execution and MI in both tasks. Thus, this paradigm provides an opportunity to investigate writing and drawing movements for instance in patients with WC by avoiding confounding dystonic co-contractions.

## Data Availability Statement

The original contributions presented in the study are included in the article/Supplementary Material, further inquiries can be directed to the corresponding author.

## Ethics Statement

The studies involving human participants were reviewed and approved by the Ethics Committee of the University of Kiel. The participants provided their written informed consent to participate in this study.

## Author Contributions

AB, IT, AK, and KZ contributed to the conception and design of the study. AB, IT, CG, and AK organized the database. AB, IT, CG, and OG performed the statistical analysis. AB, IT, and KZ wrote the first draft of the manuscript. AK wrote sections of the manuscript. All authors contributed to manuscript revision, read, and approved the submitted version.

## Conflict of Interest

AB received an intramural research grant from the Department of Neurology, Kiel. CM is the owner and author of the SCWin analysis software. KW served as a consultant for BIAL outside the present work. KZ has received research support from Strathmann and the German Research Council. She reports speaker’s honoraria from Bayer Vital GmbH, BIAL, AbbVie Allergan, and Merz outside the submitted work. She has served as a consultant and received fees from Merz, Ipsen, Alexion, and the German Federal Institute for Drugs and Medical Devices (BfArM). The remaining authors declare that the research was conducted in the absence of any commercial or financial relationships that could be construed as a potential conflict of interest.

## Publisher’s Note

All claims expressed in this article are solely those of the authors and do not necessarily represent those of their affiliated organizations, or those of the publisher, the editors and the reviewers. Any product that may be evaluated in this article, or claim that may be made by its manufacturer, is not guaranteed or endorsed by the publisher.

## Footnotes

1
http://www.fil.ion.ucl.ac.uk
