# Supplementary material for: Neural Correlates of Executed Compared to Imagined Writing and Drawing Movements: A Functional Magnetic Resonance Imaging Study
Source: Front Hum Neurosci. 2022 Mar 18;16:829576. doi: 10.3389/fnhum.2022.829576 (PMC8973008; doi:10.3389/fnhum.2022.829576)
Supplement: Supplementary file 1 [file Data_Sheet_1.pdf]

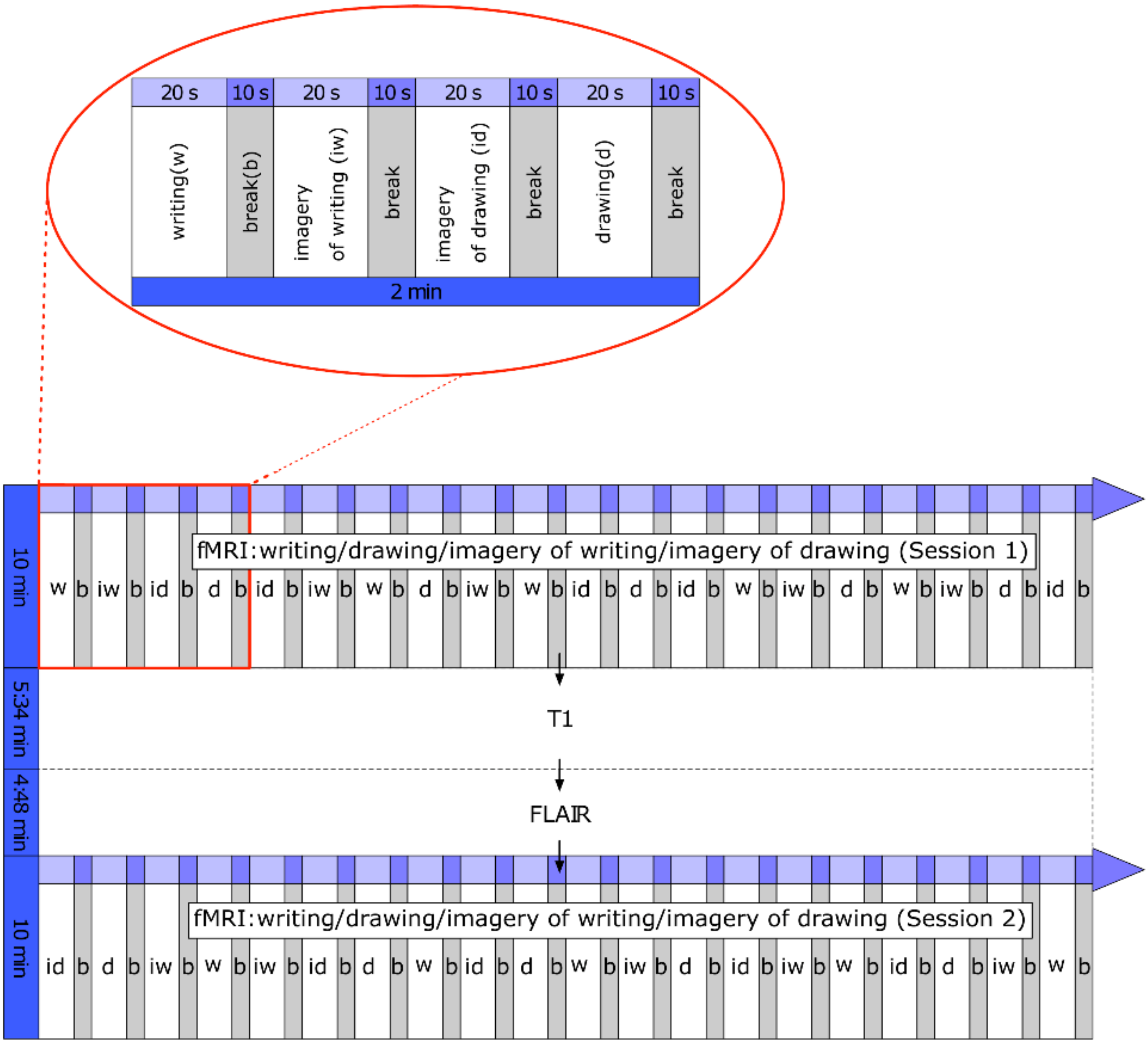

Supplementary Figure 1. Timeline of the fMRI paradigm. The figure depicts the exact process of both sessions of the fMRT paradigm with the anatomical image acquisition in between.
