# Supplementary material for: Neural Correlates of Executed Compared to Imagined Writing and Drawing Movements: A Functional Magnetic Resonance Imaging Study
Source: Front Hum Neurosci. 2022 Mar 18;16:829576. doi: 10.3389/fnhum.2022.829576 (PMC8973008; doi:10.3389/fnhum.2022.829576)
Supplement: Supplementary file 2 [file Table_1.pdf]

Supplementary Table 1. *Definition of the regions of interests for the region of interest analysis. The areas correspond to the significant peaks from the main ALE analysis published by Planton et al. (2013). In the case of no corresponding AAL region or a selectable region in the WFU PickAtlas, we defined the respective ROI as a sphere of 10mm around the peak coordinate (Planton et al., 2013; Table 2). As there is an intersection of 32 voxels between the stated definitions of area (2) and (3), the size of the aggregate mask used for the ROI-analysis is smaller than the sum of the sizes of the listed areas.*

**Area**	**Definition**	**Voxel count**	**Volume (mm³)**
(1) Gyrus frontalis medius - left	Sphere with r = 10mm around [-22, -8, 54]	515	4120
**(2) Primary motor cortex & Somatosensory cortex - left**	Brodmann Areas 1 -4 – according to the WFU PickAtalas	5139	41110
(3) Superior parietal lobule - left	AAL label “Parietal_Sup_L”	2065	16520
**(4) Supplementary and pre-supplementary motor area - left**	AAL label “Supp_Motor_Area_L”	1808	14460
(5) Anterior cerebellar lobe - right	anterior cerebellar lobe – according to the WFU PickAtalas	3530	28240
(6) Thalamus - left	AAL label “Thalamus_L”	1009	8072
**(7) Ventral premotor cortex & inferior frontal gyrus - left**	Sphere with r = 10mm around [-50, 6, 26]	515	4120
(8) Posterior cerebellum - right	posterior cerebellar lobe – according to the WFU PickAtlas	8636	69090
(9) Gyrus frontalis medius - right	Sphere with r = 10mm around [26, 0, 54]	515	4120
(10) Inferior parietal lobule - right	AAL label “Parietal_Inf_R”	1345	10760
**(11) Posterior inferior temporal cortex - left**	Sphere with r = 10mm around [-46, -62, -12]	515	4120
(12) Putamen - left	AAL label “Putamen_L”	1090	8720
