# Supplementary material for: Neural Correlates of Executed Compared to Imagined Writing and Drawing Movements: A Functional Magnetic Resonance Imaging Study
Source: Front Hum Neurosci. 2022 Mar 18;16:829576. doi: 10.3389/fnhum.2022.829576 (PMC8973008; doi:10.3389/fnhum.2022.829576)
Supplement: Supplementary file 3 [file Table_2.pdf]

AAL label	Side	MNI coordinates of cluster peak			t value of cluster peak	p value of local cluster peak*	Cluster size	p value on cluster level
		x	y	z				
**A** Whole brain analysis								
Postcentral	L	-33	-31	52	8.65	0.005	2939	0.000
Occipital_Mid	L	-21	-88	9	7.70	0.015	690	0.000
Cerebellum VIII	R	27	-64	-47	7.54	0.018	1168	0.000
Cerebellum IV V	*R*	*9*	*-55*	*-14*	*7.30*	*0.024*		
Parietal_Inf	R	45	-34	49	5.08	0.365	359	0.001
**B** ROI analysis								
Putamen	L	-27	-4	3	6.32	0.018	167	0.006
Precentral	L	-51	2	26	5.64	0.047	97	0.026

Supplementary Table 2. *Main effects of writing. The AAL labels of the cluster peaks on whole brain level (A) are reported together with the cluster size and the t value of the respective cluster peak and the cluster p-value ($p < 0.05$, FWE corrected). Further relevant local cluster peaks within a cluster are printed in italics. In section (B) the clusters peaks are listed, that became significant on $p < 0.05$ (FWE corrected) on cluster level in the ROI analysis (see supplementary table 1).*
