# Supplementary material for: Neural Correlates of Executed Compared to Imagined Writing and Drawing Movements: A Functional Magnetic Resonance Imaging Study
Source: Front Hum Neurosci. 2022 Mar 18;16:829576. doi: 10.3389/fnhum.2022.829576 (PMC8973008; doi:10.3389/fnhum.2022.829576)
Supplement: Supplementary file 4 [file Table_3.pdf]

AAL label	Side	MNI coordinates of cluster peak			t value of cluster peak	p value of local cluster peak*	Cluster size	p value on cluster level
		x	y	z				
**Conjunction: Writing execution + writing imagery**								
Fusiform	R	33	-67	-17.2	7.0	0.000	39	0.000
Cerebellum_8	R	27	-67	-46.9	6.1	0.002	17	0.004
Fusiform	L	-42	-67	-17.2	6.1	0.002	14	0.005
Putamen	L	-21	5	5.9	5.3	0.022	6	0.013
**Conjunction: Drawing execution and drawing imagery**								
Putamen	L	-21	8	5.9	5,7	0.006	19	0.003
Cerebellum_8	R	27	-64	-46.9	5,6	0.009	10	0.008
Cerebellum_6	R	33	-67	-20.5	5,2	0.028	3	0.022
SMA	L	-6	-10	62	4.98	0.048	1	0.034
**Conjunction: Writing imagery + drawing imagery**								
Cerebellum_8	R	27	-64	-47	5.57	0.009	11	0.007
Putamen	L	-21	5	5.9	5.267	0.022	6	0.013
Cerebellum_6	R	33	-67	-20.5	5.17	0.028	3	0.022
**Conjunction: Writing execution + drawing execution**								
Precentral	L	-39	-16	52	9.946	0.000	736	0.000
Vermis_4_5		6	-55	-13.9	9.919	0.000	665	0.000
Precentral	L	-57	8	22.4	7.469	0.000	59	0.000
Putamen	L	-21	11	9.2	7.049	0.000	139	0.000
Supp_Motor_Area	L	-6	-10	55.4	6.738	0.000	56	0.000
Thalamus	L	-15	-19	2.6	6.310	0.001	27	0.000
Cerebellum_Crus1	L	-42	-67	-20.5	5.948	0.003	10	0.008
Parietal_Inf	R	48	-40	48.8	5.088	0.035	6	0.013
Paracentral_Lobule	L	-6	-13	75.2	5.078	0.036	2	0.027
Cerebellum_Crus1	R	45	-55	-27.1	5.020	0.043	1	0.034

Supplementary Table 3*. Results of the conjunction contrasts within the ROI analysis. Clusters and cluster peaks are significant within the ROI analysis after FWE-correction on $p < 0.05$.*
